# Filicide Beyond Maternal Motives, a Forensic Psychiatry Unit-Based Study in Sri Lanka

**DOI:** 10.1192/bjo.2026.11097

**Published:** 2026-06-30

**Authors:** Amodha Medagedara, Charitha Suriyaarachchi, BDJV Peris, LP Vidyatilake, CTK Fernando

**Affiliations:** 1 ELFT, Luton, United Kingdom; 2 Faculty of Medicine, University of Kelaniya, Ragama, Sri Lanka; 3 PGIM, Colombo, Sri Lanka; 4 NIMH, Angoda, Sri Lanka

## Abstract

**Aims:**

Though the motives behind filicide had been extensively studied the factors that sow the seeds of those motives and how to take their edges off had rarely been investigated thus far in Sri Lanka and worldwide at large.Thus the aim of this study is to describe the demographic and clinical characteristics and associated factors in female patients referred following filicide to National Institute of Mental Health, the largest in patient forensic psychiatry facility in Sri Lanka over a period of 12 years.

**Methods:**

Data was collected from the bed head tickets (BHTs) and descriptive statistics were analysed.

**Results:**

Total of 41 BHTs were examined. 92.7% of the mothers were aged 18–40 years, 78.7% were educated up to grade 11 or below. 61.1% were from rural areas. 17.1% wereemployed. 36.6% had been diagnosed with a psychiatric illness prior to the event with depression (31.3%) being the commonest. 75.6% had not been on treatment. 73.1% of the undiagnosed, had demonstrated symptoms of psychiatric illnesses but had been diagnosed only following the filicide. Harmful use of substances was evident in 44.7% of the fathers and 7.9% doubted the paternity. 61.1% of the mothers had been subjected to domestic violence by their husbands and 63.6% had considered the support received from husbands to be inadequate. Monthly income was <50,000 in 100%. 30.8%, 41% and 25.6% of victims were aged <24 hours, 0–1 year and 1–5 year respectively. 59.5% pregnancies were unplanned. 30.8% had attempted abortions. 28.9% had had home deliveries. 21.1% of the victims had had post-natal complications. 32.4% of the mothers had attempted suicide and 22.2% had attempted homicide of the victim. 23.7% of the victims had been abused; with mother (62.5%) being the commonest abuser. Commonest modes of filicide were drowning (42.9%) and strangulation (28.6%).

**Conclusion:**

Beyond provision of the blanket defence and analysis of the maternal motives, prevention of filicide requires refining of several individual and social factors that have been overlooked.

